# The Evolution of Ketosis: Potential Impact on Clinical Conditions

**DOI:** 10.3390/nu14173613

**Published:** 2022-09-01

**Authors:** Latha Nagamani Dilliraj, Giovanna Schiuma, Djidjell Lara, Giovanni Strazzabosco, James Clement, PierPaolo Giovannini, Claudio Trapella, Marco Narducci, Roberta Rizzo

**Affiliations:** 1 Department of Chemical, Pharmaceutical and Agricultural Sciences, University of Ferrara, 44121 Ferrara, Italy; 2 BetterHumans, Inc., 3653 NE 77th Avenue, Gainesville, FL 32609, USA

**Keywords:** beta-hydroxybutyrate, evolution, ketogenesis, anti-inflammatory

## Abstract

Ketone bodies are small compounds derived from fatty acids that behave as an alternative mitochondrial energy source when insulin levels are low, such as during fasting or strenuous exercise. In addition to the metabolic function of ketone bodies, they also have several signaling functions separate from energy production. In this perspective, we review the main current data referring to ketone bodies in correlation with nutrition and metabolic pathways as well as to the signaling functions and the potential impact on clinical conditions. Data were selected following eligibility criteria accordingly to the reviewed topic. We used a set of electronic databases (Medline/PubMed, Scopus, Web of Sciences (WOS), Cochrane Library) for a systematic search until July 2022 using MeSH keywords/terms (i.e., ketone bodies, BHB, acetoacetate, inflammation, antioxidant, etc.). The literature data reported in this review need confirmation with consistent clinical trials that might validate the results obtained in in vitro and in vivo in animal models. However, the data on exogenous ketone consumption and the effect on the ketone bodies’ brain uptake and metabolism might spur the research to define the acute and chronic effects of ketone bodies in humans and pursue the possible implication in the prevention and treatment of human diseases. Therefore, additional studies are required to examine the potential systemic and metabolic consequences of ketone bodies.

## 1. Introduction

Ketone bodies are small compounds created from fatty acids that serve as an alternative mitochondrial energy source when insulin levels are low, such as during fasting or strenuous exercise [1].

The most important ketone bodies in humans are acetoacetate (AcAc) and β-hydroxybutyrate (BHB), in particular, the R-enantiomer of BHB. Ketone bodies are believed to be adaptive molecules secreted by the liver and quickly distributed to vital organs as a part of an integrated survival mechanism evolved and conserved to provide bioenergetic and signaling advantages when humans face life-threatening conditions or risk factors that could increase the likelihood of premature death [2]. During times of scarce glucose, for example, during fasting or strenuous exercise, BHB is the currency by which energy stored in adipose tissue is turned into fuel that serves the cells to maintain their functions. BHB derives from fatty acids mobilized from adipose tissue and transported to the liver. BHB circulates in the blood to all tissues. After being absorbed into a cell, BHB is broken down in the mitochondria to generate acetyl-CoA, which is further metabolized into ATP. This is the canonical “energy currency” function of BHB. 

By reducing carbohydrate ingestion, there is an exhaustion of the body’s glucose reserve, shifting the metabolism into ketogenesis, inducing hepatic oxidation of fatty acids, and producing ketones as an important alternative to glucose as the body’s energy source [3]. Ketosis is a physiological metabolic state characterized by an increased serum ketone body level from ~0.2 mM to above 3.0 mM [4], caused by exercise [5], fasting/starvation [6], or diabetes [7,8]. In peculiar conditions, the ketosis might develop into overt ketoacidosis, with a decreased serum bicarbonate level and pH, causing serious illness and hospitalization. Ketoacidosis is mainly associated with alcoholism and diabetes mellitus type I, starvation, particularly during malnutrition, and poor dietary intake in people following low-carbohydrate and/or low-caloric diets [9]. 

In addition to the metabolic function of ketone bodies, they also have several signaling functions separate from energy production. Ketone bodies are involved in epigenetic changes [10,11,12], controlling cellular signaling metabolites [13], gut microbiota, and butyrogenesis [14]. The epigenetic changes regulate cellular gene expression and metabolism, with an implication for physiological and pathological conditions [15,16] 

In this review, we discuss not only the metabolic effect of ketone bodies but also their implication in clinical conditions, suggesting possible future research fields in the use of these molecules as a clinical approach. 

## 2. Methods

In this perspective, we have reviewed the main current data referring to ketone bodies in correlation with nutrition and metabolic pathways as well as to signaling functions, and the potential impact on clinical conditions. Data were selected following eligibility criteria according to the reviewed topic. We used a set of electronic databases (Medline/PubMed, Scopus, Web of Sciences (WOS), Cochrane Library) for a systematic search until July 2022 using MeSH keywords/terms, such as “ketone bodies”, “BHB”, “acetoacetate”, “inflammation”, “anti-oxidant”, “critical illness”. We applied no date or language restrictions. We followed the Preferred Reporting Items for the Systematic Review and Meta-Analysis (PRISMA) statement [17]. Two independent reviewers performed title-abstract screening on all selected studies, then the full texts of the selected articles were reviewed. In cases of duplicate information, the data were checked and combined. Studies reporting ketone bodies as well as BHB and acetoacetate were selected. Publications were selected using specific keywords (i.e., ketone bodies, BHB, acetoacetate, inflammation, antioxidant, etc.) also according to the date of publication (not older than 1990) and for the fulfillment of the topic of this review. Studies that were just case reports and commentaries were excluded. The extraction of the data from included studies was performed by two reviewers separately, considering key characteristics including publication year, author, type of study, country, sample size, and laboratory findings. The funnel plot and Egger’s regression test were used to assess publication bias [18].

## 3. Metabolism of Ketones: Ketogenesis and Ketolysis

**Figure 1:**
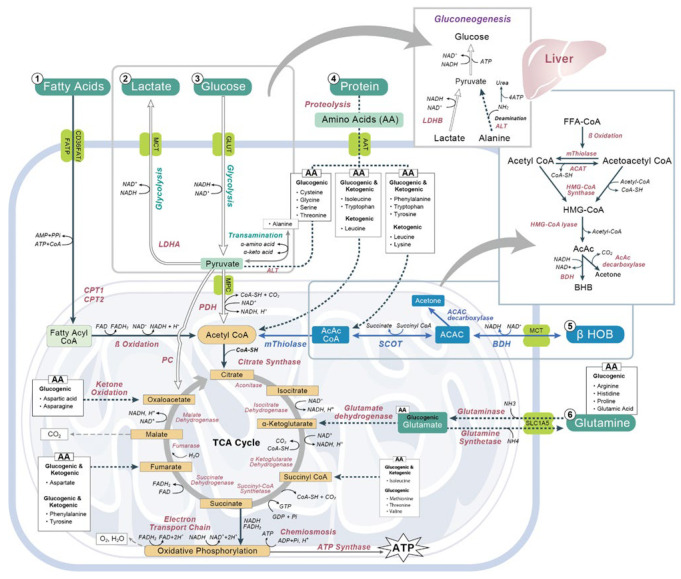
Schematic Diagram of the metabolic pathways of key energy sources in the human body. NAD: nicotinamide adenine dinucleotide; FADH: reduced flavin adenine dinucleotide; NADH: nicotinamide adenine dinucleotide (NAD) + hydrogen (H); PDH: pyruvate dehydrogenase; LDH: lactate dehydrogenase; CPT: carnitine palmitoyl transferase; ß HOB: beta hydroxybutyrate; BDH: D-3-hydroxybutyrate dehydrogenase; SCOT: succinyl-CoA acetoacetate transferase; AcAc: acetoacetate; CoA: coenzyme A; CoA-SH: coenzyme A with sulfhydryl functional group; CO2: carbon dioxide; H2O: water; ADP: adenosine diphosphate; ATP: adenosine triphosphate; Pi: phosphorylated forms of phosphatidylinositol; NH3: ammonia; NH4: ammonium; H+: hydrogen ion; HMG-CoA: ß-hydroxy-ß-methylglutaryl-CoA; MCT: monocarboxylate transporters; GLUT: glucose transporter; AAT: amino acid transporter; ALT: alanine amino transferase; PC: pyruvate carboxylase; MPC: mitochondrial pyruvate carrier.

The human body produces energy as ATP generated by the mitochondria, for survival. The main energy sources are carbohydrates, fats, amino acids (predominantly glutamine), lactate, and ketone bodies (Figure 1).

Ketone bodies, that are produced during ketogenesis are of three types: acetone, acetoacetate (AcAc), and 3-hydroxybutyrate (BHB). The mainly produced ketone body molecule during ketogenesis is BHB, mainly the R-BHB enantiomer. BHB levels increase in plasma much faster than acetoacetate or acetone, for example, in prolonged fasting [19]. During the conditions such as fasting or vigorous exercise, the consumption of blood glucose lowers insulin levels, turning on ketogenesis, and triglycerides are catabolized to fatty acids that are converted into ketone bodies in the liver [2]. Ketone bodies arrive in metabolically active tissues (muscle, brain) via the blood stream to be metabolized into acetyl-CoA and eventually ATP (Figure 1). BHB is converted, in extrahepatic tissues, by the enzyme 3-hydroxybutyrate dehydrogenase (BDH1) to AcAc [2], which generates Acetyl-CoA [20,21] by exchanging the CoA-fraction from succinyl-CoA [22] by succinyl CoA-oxoacid transferase (SCOT) [23]. Acetyl-CoA enters the TCA cycle and produces 22 ATP per molecule post oxidative phosphorylation. Acetoacetate can be converted also to 3-hydroxybutyrate by BDH or to acetone via a non-enzymatic decarboxylation [20]. 

The short-chain fatty acids (SCFAs), butyrate, acetate, and propionate (in a molar ratio of 3:1:1), are produced as microbial fermentative end-products of undigested/unabsorbed dietary carbohydrates [24,25]. Butyrate is produced by acetate and/or lactate-utilizing butyrate-producing bacteria through the butyryl-CoA: acetate CoA-transferase pathway [24,26,27]. This pathway is typically present in Firmicutes, within Lachnospiraceae (*Eubacterium hallii*, *Eubacterium rectale*, *Coprococcuscatus*, *Roseburia intestinalis*), Ruminococaceae (*Faecalibacterium prausnitzii*), and *Clostridium* spp. in which butyrate and acetyl-CoA are formed from butyryl-CoA and the transformation of the CoA moiety to the external acetate molecule [25,26,27,28,29], while *Bifidobacterium* and *Lactobacillus* spp. use lactate to produce SCFAs. These molecules, similarly, and in combination with ketone bodies, have an anti-inflammatory role via an epigenetic mechanism such as butyrate-associated HDAC inhibition [30].

## 4. Endogenous Sources of Ketone Bodies

**Figure 2:**
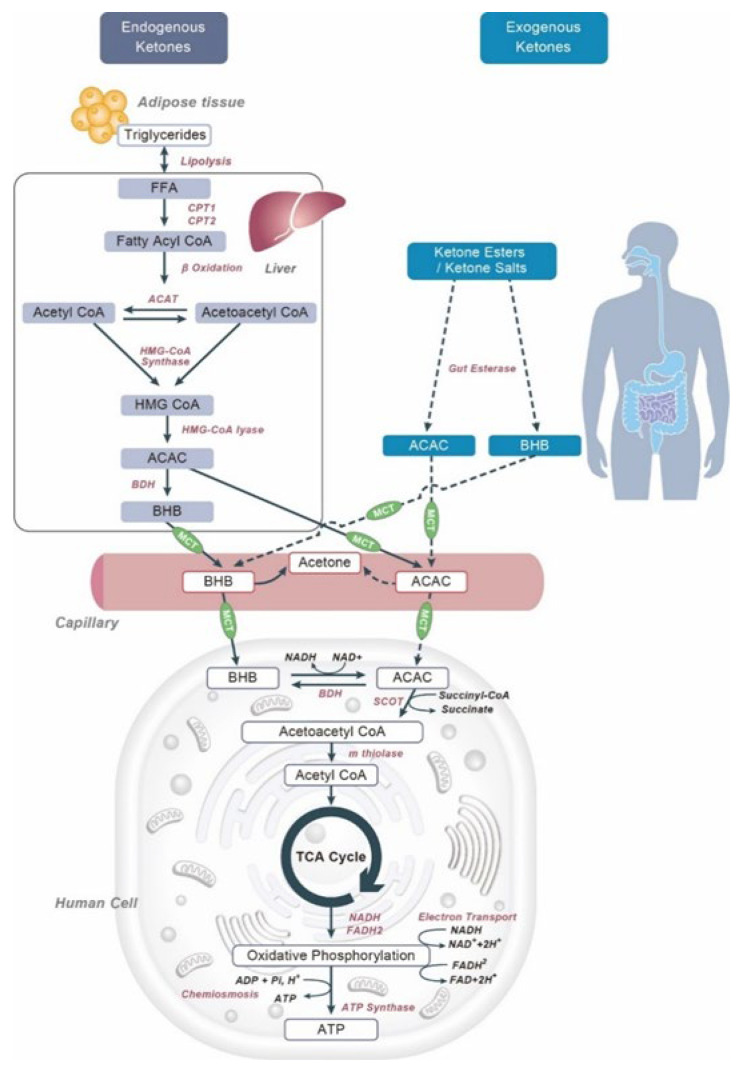
Endogenous and exogenous metabolic pathways. NAD: nicotinamide adenine dinucleotide; FADH: reduced flavin adenine dinucleotide; NADH: nicotinamide adenine dinucleotide (NAD) + hydrogen (H); CPT: carnitine palmitoyl transferase; ß HOB: beta hydroxybutyrate; BDH: D-3-hydroxybutyrate dehydrogenase; SCOT: succinyl-CoA acetoacetate transferase; AcAc: acetoacetate; CoA: coenzyme A; ADP: adenosine diphosphate; ATP: adenosine triphosphate; Pi: phosphorylated forms of phosphatidylinositol; H+: hydrogen ion; HMG-CoA: ß-hydroxy-ß-methylglutaryl-CoA; MCT: monocarboxylate transporters.

Ketone bodies might derive from endogenous or exogenous sources (Figure 2): endogenous ketones are normally present in our bloodstream and are produced mostly by the liver and by certain species of gut bacteria.

Microbiota-derived SCFAs, primarily butyrate, acetate, and propionate are metabolites produced by gut microbiota via dietary non-digestible carbohydrate fermentation [24,25]. SCFAs play a significant role in CHO and lipid metabolism. Butyrate and acetate are used as precursors for lipid synthesis (cholesterol, long-chain fatty acids), whereas propionate is used as a precursor for hepatic gluconeogenesis [24,31]. SCFAs are influenced by the diet (fiber, fats, plant-based proteins) and are important during pregnancy and lactation, controlling the formation of infant gut microbiota. The difference in SCFA-producing bacteria in gut microbiota leads to a different anti-inflammatory state with an impact on inflammatory conditions (e.g., obesity, asthma) [11,12]. 

In physiology, ketosis can be achieved by fasting, via exogenous supplementation, or the consumption of a ketogenic diet. Fasting from 12–16 h up to 48 h increases serum ketone body concentrations but may elicit inconsistent effects on performance in humans and animal models [32].

## 5. Exogenous Sources of Ketone Bodies

Exogenous ketone bodies are able to obtain ketosis [1]. Exogenous ketone bodies can be acquired from diet or supplements, such as medium-chain triglyceride (MTC) oils, ketone salts, or esters. MTCs are usually sold as oils or as lyophilized powders and are used to provide the mitochondria with non-carbohydrate energy. They are composed of 8–10 carbon fatty acid chains and they are capable of inducing ketosis thanks to the excess of acetyl CoA produced by the liver while it metabolizes MTC. Ketone salts can be a good alternative to ketogenic diets thanks to their application versatility. They are easy to take and can quickly raise the blood level of ketones. These compounds have a low risk of health issues in humans, and the only concern happens because of the sodium salts as they could increase blood-free sodium levels [33]. Another issue could be the racemic mixture that is present in this product; usually, BHB salts are a combination of D-BHB and L-BHB. It is known that the L form cannot be metabolized by the liver and remains in the blood until it is eliminated through urine or feces. Ketone esters are produced by synthesis, linking one molecule of BHB. Ketone esters are more capable of increasing and maintaining the state of ketosis compared with ketone salts. Some small clinical trials have compared the two different forms of the same molecule, concluding that the ketone esters were able to increase free BHB levels 50% higher than ketone salts [34]. Recently, supplementation with ketone body esters has shown an improvement in exercise performance [35,36]. 

A ketone diet is characterized by the supply of approximately 80–90% of calories from fat, 10–15% of calories from protein, and <5% calories from carbohydrates [37], stimulating fat oxidation and promoting fat loss, which are important in obesity conditions [38]. The ketone diet has demonstrated successful results in the treatment of epilepsy and other neurological conditions [39]. However, prolonged ketone diets seem to have an impact on liver steatosis [40,41], glucose homeostasis [40,41,42], and dyslipidemia [43]. 

## 6. Metabolic Functions of Ketone Bodies in Vital Organs

During low-carbohydrate conditions, ketone bodies are used in proportion to their plasma concentrations, and consequently, liver production. 

It has been demonstrated that the acetoacetate generated from an oral intake of BHB esters reaches first the heart, followed by the kidneys, brain, skeletal muscles, and intestine. Normally, heart tissue can oxidize ketones which enter cardiomyocyte cells, thanks to a specific carrier called MCT1 (monocarboxylate transporter) [44]. In the mitochondria of these cardiomyocytes, ketones are converted into AcAc and then into aceto-acetyl CoA, thanks to the activity of the enzyme SCOT. After this conversion, a thiolase transforms the last product into two molecules of acetyl CoA, which enter the TCA cycle for the production of ATP. In stressful situations, such as heart failure and aortic stenosis-induced left ventricular hypertrophy, the circulating levels of ketones and their oxidation states are heightened, supporting the role of ketones in providing the increased energy demanded by the heart [45].

In the ketosis physiological state, BHB can easily enter proximal tubular cells because there is no saturation mechanism [10]. There, BHB is oxidized through the TCA cycle that produces acetyl CoA, which is then transformed via oxidative phosphorylation into ATP.

An adequate and consistent energy supply is necessary to maintain brain cell functions since low glycogen storage is present inside the brain [46]. This is evidenced in pathological conditions with defects in the brain (e.g., glucose transporter type 1 (GLUT-1 deficiency), with impaired cerebral glucose uptake and consequent seizures, movement disorders, and cognitive impairments [47]. The uptake of ketone bodies across the blood–brain barrier (BBB) is possible via monocarboxylate transporter (MCT) [48], with MCT1-isoform expressed by endothelial cells and astroglia [9]. The brain uses ketone bodies, which can give more than 50% of its energy requirements. 

Ketone bodies are also important energy substrates for skeletal muscle, with a robust anticatabolic response, reducing phenylalanine efflux from muscle [49].

Ketone bodies derived from short-chain fatty acids are employed by colonic epithelial cells as respiratory fuels where they predominantly use butyrate sequentially followed by acetoacetate, glutamine, and glucose, notwithstanding the interaction of substrates [50]. Ketone body signaling facilitates Lgr5+ intestinal stem cell (ISC) homeostasis aiding in post-injury intestinal regeneration, restoring intestinal regeneration, and resuming ISC function [51]. 

## 7. Biological Properties of Ketone Bodies

**Figure 3:**
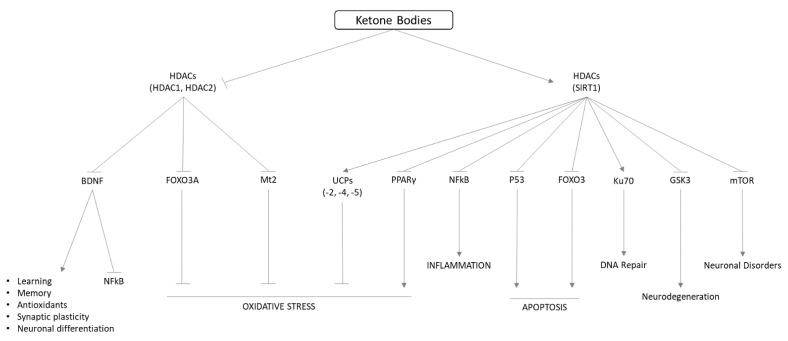
Molecular pathways involved in ketone bodies effect on oxidative stress, inflammation, and epigenetic control. HDAC: histone deacetylase; BDNF: brain-derived neurotrophic factor; NFkB: nuclear factor kappa-light-chain-enhancer of activated B cells; FOXO3A: forkhead box O3; Mt2: mammalian metallothionein-2; UCP: uncoupling protein; PPARγ: peroxisome proliferator-activated receptor γ; p53: protein 53; Ku70: DNA repair subunit protein; GSK3: serine/threonine protein kinase; mTOR: mammalian target of rapamycin.

The increase in ketone body blood levels derives from fatty acid breakdown during low carbohydrate availability, resulting in a danger signal of starvation and a physiological response for improving survival during starvation. The energy production from ketone bodies is associated with increased radical oxygen species (ROS) release in the mitochondria, an increase in NAD+ levels, and a lower AMP/ATP ratio. Ketone bodies and the ketogenic diet act in upregulating anti-oxidant and anti-inflammatory mechanisms (Figure 3).

Consistently, ketone bodies preserve mitochondria and their role in cellular energy homeostasis [52]. An increased uptake of Ca2+ into mitochondria enhances ROS production and blocks the synthesis of ATP, inducing cytochrome c release and mitochondrial membrane potential [53]. These modifications cause mitochondrial swelling and apoptosis [54], with consequently impaired energy homeostasis [55]. BHB is able to maintain mitochondrial density and function [56] by controlling the mitophagy of damaged mitochondria and inducing the renewal of the mitochondria population [57]. The mitochondrial biogenesis is enhanced via Nrf2 activation, which induces the transcription of PGC-1 (peroxisome proliferator-activated receptor gamma coactivator-1) [58], which controls the transcription of TFAM (mitochondrial transcription factor A), resulting in the replication of mitochondrial DNA and the mitochondrial biogenesis [59].

The block of glutathione peroxidase (GSH-Px), a key rate-limiting enzyme in ROS formation [60] by BHB, reduces the production of ROS, with a consequent improvement in mitochondrial respiration and homeostasis [61,62,63,64,65], ATP production, activation of adenosine receptors that lower oxidative stress [66], upregulation of antioxidant genes and activation of antioxidant enzymes that control lipid peroxidation and protein oxidation [67]. 

Ketones’ anti-inflammatory effects are related to the inhibition of the NLRP3 (NOD-like receptor pyrin-domain containing-3) inflammasome, which activates caspase-1 and the release of the pro-inflammatory cytokines (IL-1β and IL-18) [68]. Ketone bodies block the K+ efflux, which activates the NLPR3 inflammasome [69]. During brain injury induced by the middle cerebral artery occlusion (MCAO) model, ketone bodies inhibited NLRP3 and inflammation [70]. In a randomized, controlled dietary intervention trial with 40 overweight subjects aged 18–55 years fed with a diet very low in carbohydrates or an isocaloric diet low in fat for 12 weeks, the subjects following the ketogenic diet showed lower inflammation, with the reduction of interleukin-8 (IL-8), TNF-alpha, plasminogen activator inhibitor-1 (PAI-1), monocyte chemoattractant protein (MCP-1), E-selectin, and intercellular adhesion molecule-1 (ICAM-1) in the presence of mild inflammation [71]. 

The immune response is controlled by ketones by the binding of BHB to HCAR2 (hydroxycarboxylic acid receptor 2), which results in the induction of prostaglandin D2 (PGD2) production by cyclooxygenase 1 (COX1) [72] and the inhibition of NF-KB (nuclear factor kappa-light-chain-enhancer of activated B cells) mediated inflammation through the blockage of IKB kinase (IKK), by a metabolite of PGD2. 

BHB is involved in controlling cellular function via epigenomic regulation. Ketone bodies control histone post-translational modifications, including histone methylation (Kme), histone/lysine acetylation (Kac), and β-hydroxybutyrylation (Kbhb), which regulate chromatin architecture and gene expression. BHB is able to inhibit the histone deacetylase (Class 1 and Class IIa HDACs) leading access to transcription factors of genes encoding oxidative stress resistance factors like FOXO3 (forkhead box O3) and Mt2 (Mammalian metallothionein-2) [73]. The induction via BHB of histone Kbhb levels with site-specific lysine residues (H3K4, H4K8, H3K9, H4K12, H3K56) is increased in human embryonic kidney 293 (HEK293) cells during prolonged fasting, supporting a direct role in chromatin structure and functions regulation [74]. In HEK293 cells transiently transfect with ORM (yeast)-Like protein isoform 3 (ORMDL3) mRNA expression, an asthma susceptibility gene [75], BHB controlled inflammation inhibiting ER stress response pathway proteins and enhancing both Foxp3 and manganese superoxide dismutase (MnSOD) transcription via AMP-activated protein kinase (AMPK) activation, leading to a decrease in cellular oxidative stress [76]. 

BHB inhibition of HDACs leads to increase expression of brain-derived neurotrophic factor (BDNF), which exerts neuroprotective effects against various insults to the central nervous system, as the functional recovery after traumatic brain injury (TBI) in mice [77].

BHB can act as an inducer of transcription factor Nrf2 (nuclear factor-erythroid factor 2-related factor 2). Nrf2 is a transcription factor that regulates the cellular defense against toxic and oxidative insults through the upregulation of the expression of genes involved in the oxidative stress response and drug detoxification [78]. Human microvascular endothelial cells (HMEC-1) exposed to ketone bodies increased NRF2 expression with a clear translocation to the nucleus and induction of antioxidant proteins [79]. 

## 8. Role of Ketone Bodies in Pathophysiological Ailments

Critical illness has demonstrated various disruptions in metabolism and mitochondrial function. Whether it arises from organ failure or microbial infections, the metabolic response to these ailments requires maintenance of metabolic balance, nutrient utilization for different activities, and functional recovery. On the other hand, a metabolic response can create clinical consequences such as catabolic processes that can impair physiological stability. Furthermore, a long period of metabolic imbalance could produce mitochondrial dysfunction, including energy crisis and high free-radical production, resulting in a compromised immune system, tissue and organ failure, and death [80,81,82,83]. 

Critical-illness-affected patients require an energy source to support physiological stress responses and to give robust protection to critical organs such as the heart, brain, liver, and kidney [81,83] (Figure S1). Additionally, increasing evidence across animal and human studies has shown ketones are a beneficial alternative substrate due to reloading acetyl-coenzyme-A through an independent pathway irrespective of glucose levels [84], and are useful to maintain the cytosolic NAD+ (nicotinamide adenine dinucleotide) pool which is pivotal for cellular survival, antioxidants, and pro-survival pathways [85]. As serum ketone body concentration varies during physiological and pathological conditions and acts as potent endogenous signaling molecules, they may act in cellular protection and repair, mitochondrial biogenesis, antioxidant defenses, and enhanced autophagy [80,82,86,87,88]. With the known and possible mechanistic properties of ketones, their use as an individual or adjunct therapy for different conditions in critical illness has been explored.

### 8.1. Heart Failure

During heart failure, the heart undergoes a metabolic switch favoring ketone metabolism in cardiomyocytes, which are more efficiently used than in the normal heart. Moreover, ketone body oxidation is a more efficient energy substrate than terminal fatty acid oxidation [88]. Significant improvement in the cardiac output of about 24% was observed after ketone infusion in both chronic heart failure patients and animal models with heart failure [84,89]. These cardioprotective properties can be attributed to their increased utilization in the heart or upregulation of crucial oxidative phosphorylation mediators [44]. It has also been demonstrated that ketones improve blood lipid profile in obese adults by lowering LDL (low-density lipoprotein) cholesterol and raising HDL (high-density lipoprotein) cholesterol, reducing adipocyte cell volume, and lowering serum lipolytic products [84]. Ketones’ mechanistic action in inhibiting NLRP3 inflammasome and activation of the GPCR109A (G-protein-coupled receptor) has been shown to rescue heart failure by reducing mitochondrial hyperacetylation, resulting in lower inflammation and oxidative stress and preventing atherosclerosis [84]. A study in mice also showed that BHB reduces sympathetic outflow and lowers heart rate and total energy expenditure by antagonizing GPR41, a G-protein−coupled receptor for short-chain fatty acids [88].

### 8.2. Kidneys and Liver Diseases

During the development of kidney diseases, impaired lipolysis and mTORC1 (mammalian target of rapamycin complex 1) hyperactivation are observed; with this pathological condition, ketone supplementation might be an alternative energy source for mitochondrial respiration. Moreover, ketones’ reno-protective roles are mainly via the endogenous inhibition of HDAC (histone deacetylase) and NLRP3, both increased expression of protective genes; consequently, it inhibits mTORC1, inflammation, oxidative stress, and tissue fibrosis [84,89]. Additionally, one mice study involving high-fat liver injury showed that oxidation of the ketone body acetoacetate by liver-resident macrophage-like Kupffer cells lowers fibrosis [89].

### 8.3. Brain Injury and Neuronal Diseases

Substantial evidence has shown ketone bodies’ pleiotropic neuroprotection properties due to their pivotal role in cerebral energy homeostasis and an active signaling molecule. BHB directly regulates inflammation and neurotrophic factors by inhibiting the activation of innate immune sensor NLRP3 and inhibiting HDAC, which upregulates BDNF (brain-derived neurotrophic factor) [90]. BDNF is crucial in the maintenance, restoration, and improvement of neural networks and brain functions after a brain injury. Thus, BDNF production in the brain plays an essential role in the prolonged maintenance of neuroplasticity [90]. Ketones have been known to have anti-seizure effects, which can be achieved through their action in altering synaptic neurotransmission via increasing GABA (gamma-aminobutyric acid) synthesis and decreasing glutamate synthesis [91]. Similarly, the anti-epileptic effects of ketones via activation of the KATP channels and GABA signaling lead to lower neuronal firing [92]. Multiple in vitro studies have demonstrated that ketones can increase the survival rate of cultured neocortical neurons and isolated cortical mitochondria from exposure to hydrogen peroxide both with and without glucose addition [93]. It also reduced apoptosis after hypoxia in rat hippocampal neuron cultures from various insults, including hypoglycemia, hypoxia, and N-methyl-D-aspartate-induced excitotoxicity [93]. Furthermore, ketones demonstrated strong neuroprotective properties in various animal models of brain injury, reducing neuronal apoptosis and brain edema and enhancing sensory-motor and cognitive performance [89,93]. It has also reduced neuronal loss and infarct size in animal models of stroke and reduced the glutamate release and seizure severity in a mouse model of epilepsy [89,93]. In spinal cord injury models in rats, ketosis reduced spinal lesions enhanced the GLUT 1 and MCT1 vascular transporters, and forelimb motor function improvement [94]. Preclinical studies in adult rats with moderate and severe traumatic brain injury strongly demonstrated therapeutic actions, where it showed a significant reduction of infarct and penumbral volume in MRI, decreased tissue death and edema, and improved neurological scores [91]. The administration of ketones and hypertonic saline (HTS) showed beneficial effects in managing intracranial hypertonic pressure with enhanced cerebral metabolism [95]. Thus, the administration of exogenous ketones in patients with different stages of brain injury might benefit clinical outcomes due to the suggested neuroprotective property in maintaining mitochondrial function and decreasing inflammation, oxidative stress, and seizure problems [91]. Ketogenic interventions might facilitate the brain’s utilization of ketones as an essential energy source and as a signaling molecule that may slow down the disease progression and delay or even prevent the disease onset if started earlier [96]. In vitro models of Parkinson’s disease in mouse neuronal cultures demonstrated increased cell survival, enhanced mitochondrial membrane potential, and lower cytochrome c release, while a 60% increase in cell survival was also observed in human neuroblastoma cell culture [97]. Motor function was restored and prevented losing dopaminergic neurons after several infusions of sodium BHB (1.6 mmol/kg/day) [44]. Similarly, in the in vitro model of Alzheimer’s disease, the cultured mouse hippocampal neurons were protected from amyloid beta 1 to 42 toxicity after 4 mmol/L ß-OHB was administered [44]. Ketone supplementation provided cognitive protection for several months [91] and even better performance in paragraph recall and Alzheimer’s assessment scale tests in humans subjected to a ketogenic diet [97]. While in animal models of amyotrophic lateral sclerosis, the clinical and biological manifestations of the disease were beneficially altered after exposure to hyperketonemia, which had more remarkable motor neuron survival and enhanced motor function [98].

### 8.4. Muscle Weakness

Post-recovery weakness involves more than 50 percent of patients recovering from critical illness. This condition is characterized by muscle dysfunction, atrophy, and damage, with consequent immobility, inflammation, and catabolism. The maintenance of mobility is the basis of critical care management [44]. Ketone bodies seem to have a direct effect on protein turnover, as their increase is associated with a decrease in proteins and amino acid efflux from skeletal muscle [81,99]. Additionally, ketone supplementation reduces muscle atrophy, and increased cholesterol myofiber, which is associated with muscle force [100] and stimulates muscle regeneration [81,84].

## 9. Neuroprotective Actions of Ketone Bodies

It has been observed that ketone bodies have multiple key roles in the brain, which are exerted not only during fasting but also in the newborn period. The development and function of a healthy neonatal brain appear to be related to locally produced ketones, which are the preferred precursors for fatty acids and cholesterol for the creation of dry matter in the brain [22,101,102]. Human newborns are characterized by extensive subcutaneous adipose stores [103], which provide fatty acids and ketones. The medium-chain fatty acids (MCFAs) in breast milk, synthesized de novo from glucose within the epithelium of the milk duct, promote the production of ketone bodies in the infant liver [104] and gut [105]. Some of the MCFAs in breast milk create adipose stores in the infant, that prolongs mild ketonemia after lactation ends. The energetic support of the development of the brain in infants is mainly supported by ketone uptake and oxidation, with a molar utilization of BHB that is around 50% greater than that of glucose [106,107]. The newborn rats start ketosis from the beginning of the suckling period, thanks to the MCFAs in the dam’s milk [28,108]. The levels of BHB and AcAc are 3- to 4-fold higher at the blood–brain barrier in newborns in comparison to adults [109,110]. 

### Ketone Bodies Influence Neurotransmitters

**Figure 4:**
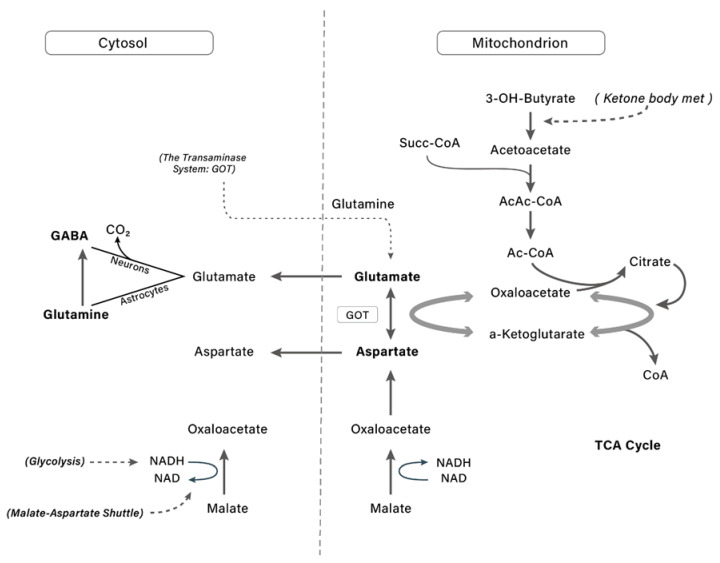
Scheme illustrating the relationship between brain metabolism of ketone bodies and that of glutamate and GABA, where the metabolism of ketone bodies of acetyl-CoA induces the increase of glutamate and GABA. 3-OH_Butyrate: β-hydroxybutyrate; Succ-CoA: succinyl-CoA; AcAc-CoA: acetoacetyl-CoA; Ac- CoA: acetyl-CoA; CoA: coenzyme A; NADH/NAD: nicotinamide adenine dinucleotide; GOT: glutamate-oxaloacetate transaminase; GABA: gamma-aminobutyric acid.

The brain’s major excitatory neurotransmitter is glutamate [111], which is not transported from blood, but is synthesized in the brain and delivered to neurons upon depolarization. Ketone bodies control neurotransmitters metabolism as acetyl-CoA production by ketone bodies decreases oxaloacetate, increasing glutamate levels and inducing GABA synthesis [112,113] (Figure 4). In the presence of ketone bodies, it metabolizes to acetyl-CoA and oxaloacetate follows citrate synthesis [110,114,115], reducing the activity of glutamic oxaloacetic transaminase (GOT) and preserving glutamate for the glutamate decarboxylase reaction to yield GABA. Ketone bodies DL-β-hydroxybutyrate were shown also to control GABA activity during the developmental, resulting in a switch from being predominantly depolarizing–excitatory to predominantly hyperpolarizing–inhibitory [116].

## 10. Discussion

This review has highlighted the impact of ketone bodies on several human physiological and pathological conditions. Ketone bodies are an alternative energy source in hypoglycemia conditions, such as when fasting or during strenuous exercise, they have several signaling functions inside human cells, affecting cell genome acetylation and consequently gene expression, controlling adipose tissue metabolism, changing sympathetic nervous system activation and the whole-body metabolic rate, inhibition, and inflammasome activation. These ketone bodies’ effects on human cells might suggest their implication in the control of human pathological conditions.

The psychophysiological and metabolic milieu that triggers the secretion of ketone bodies includes (i) starvation; (ii) severe injuries; (iii) acute infections or viral illnesses [89] (iv) physical exhaustion, and (v) in the presence of harsh ecological stressors. In these contexts, ketone bodies galvanize and modulate the body’s survival factors during these unfavorable conditions by offsetting physiological dyshomeostasis and psychophysical functionality. Therefore, the advantage of ketone metabolism is the conservation of precious glycogen reserves and the immediate supply of a potent and effective fuel for the brain. The most apparent sign that ketone bodies are a well-preserved and highly adaptive trait of evolution is the fact that even infants and embryos utilize ketone bodies as a critical bioenergetic buffer to sustain the tremendous growth of the neonatal brain. Evolutionary forces selected ketone bodies to ensure self-preservation during the most critical time for any specie evolution. Ketone bodies enter the TCA cycle with fewer steps than glucose and produce more ATP per mole than pyruvate with a lower oxygen requirement to produce more ATP per mole than glucose, preventing the depletion of NAD+ and endogenous antioxidants while increasing cellular bioenergetic efficiency [101]. Cotter et al. [117] demonstrated that postnatal mice without ketone bodies oxidation present a lethal metabolic state, even in the presence of alternative metabolic fuels supplied through milk. A similar condition seems to be present in humans, where the sudden infant death syndrome (SIDS) has been attributed to SCOT deficiency [117]. In the light of this evolutionary perspective, nutritional ketosis when induced by exogenously or endogenously seems to enhance survival during hemorrhagic shocks, severe hypoxia, cerebrovascular ischemia, heart attacks, deep wounds, traumatic brain injuries, sepsis, poisoning, and severe intoxications in in vivo animal models. Nutritional ketosis might affect the biophysical state with a possible role in controlling central fatigue, anxiety, aggression, clinical depression, sense of hunger, or perceived pain while increasing focus and mental performance. In line with seminal studies emerging from calorie restriction, more recent evidence shows that being in nutritional ketosis might control degenerative conditions including recalcitrant metabolic diseases that manifest dysfunctional homeostatic adaptations and deteriorations. 

The ketone metabolism is a constitutive feature of organ functions, mainly in the brain [118]. These findings suggest the need for clinical studies to evaluate the possible effect of the administration of exogenous ketone bodies to enhance general brain health. 

Currently, there is considerable interest in ketone body supplementation, such as drinks containing ketone esters and ketone salts, which can increase ketone bodies’ blood concentration without dietary changes [119], positively affecting the ketone body’s brain uptake and metabolism. For example, the oral ingestion of exogenous BHB is able to obtain rapid and significant ketosis (i.e., above 6 mmol/L) in humans. The oral BHB administration (3 mg KE/g of body weight) in non-fasted mice, increased acetyl-CoA and citric cycle intermediates in the brain [120], with a preferential distribution in the neocortex. Acetate supplementation increased plasma acetate and brain acetyl-CoA levels in rats [121], with no modification in brain ATP, ADP, NAD, GTP levels, or the energy charge ratio, glycogen, and mitochondrial biogenesis when compared to controls [121]. The literature data suggest that ketone bodies had a major impact on the evolution of our brains over 2 million years, when the life of hominid monkeys was characterized by intermittent starving and fat intake, optimal for the generation of ketone bodies, supporting the “ketone-brain expansion” hypothesis [122].

## 11. Conclusions

**Figure 5:**
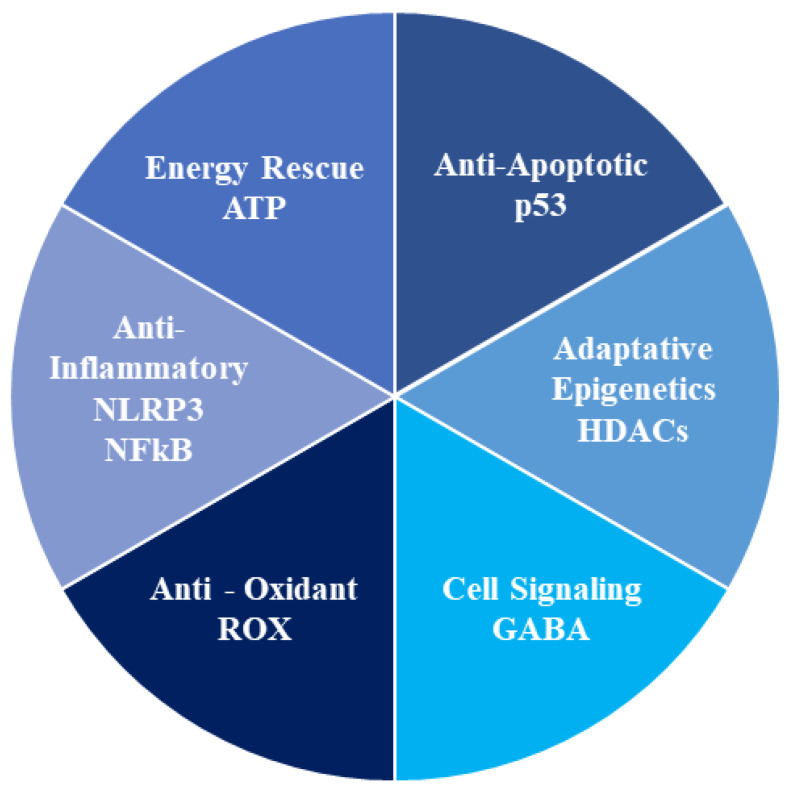
The six hallmarks of ketonic action. ATP: Adenosine triphosphate; NLRP3: NOD-, LRR- and pyrin domain-containing protein 3; NFkB: nuclear factor kappa-light-chain-enhancer of activated B cells; ROX: chemical reduction oxidation; GABA: gamma-aminobutyric acid; HDAC: histone deacetylase.

In conclusion, ketone bodies showed a significant role in controlling oxidative stress and inflammation, which result in improved mitochondrial function and growth, energy rescue, and adaptative epigenetic control (Figure 5). In this context, ketolysis is an adaptive response of the human body to resist acute and chronic diseases, acting as an alternative fuel during periods of deficient food supply, with the reduction of oxidative stress in the mitochondria, and the protection of cell functions. The absence of consistent clinical trials partially dampens the interesting results obtained in vitro and in vivo in animal models. However, the data on exogenous ketones consumption and its effect on the ketone bodies’ brain uptake and metabolism might spur the research to define the acute and chronic effects of ketone bodies in humans and pursue the possible implication in the prevention and treatment of human diseases. Therefore, additional studies are required to examine the potential systemic and metabolic consequences of ketone bodies.

## Data Availability

Not applicable.

## Supplementary Materials

The following supporting information can be downloaded at: https://www.mdpi.com/article/10.3390/nu14173613/s1. Figure S1: Target organs and pathophysiological conditions of ketone administration; possible mechanism of action as alternative fuel and signaling molecule.
