# Supplementary figures and images for: The Evolution of Ketosis: Potential Impact on Clinical Conditions
Source: Nutrients. 2022 Sep 1;14(17):3613. doi: 10.3390/nu14173613 (PMC9459968; doi:10.3390/nu14173613)

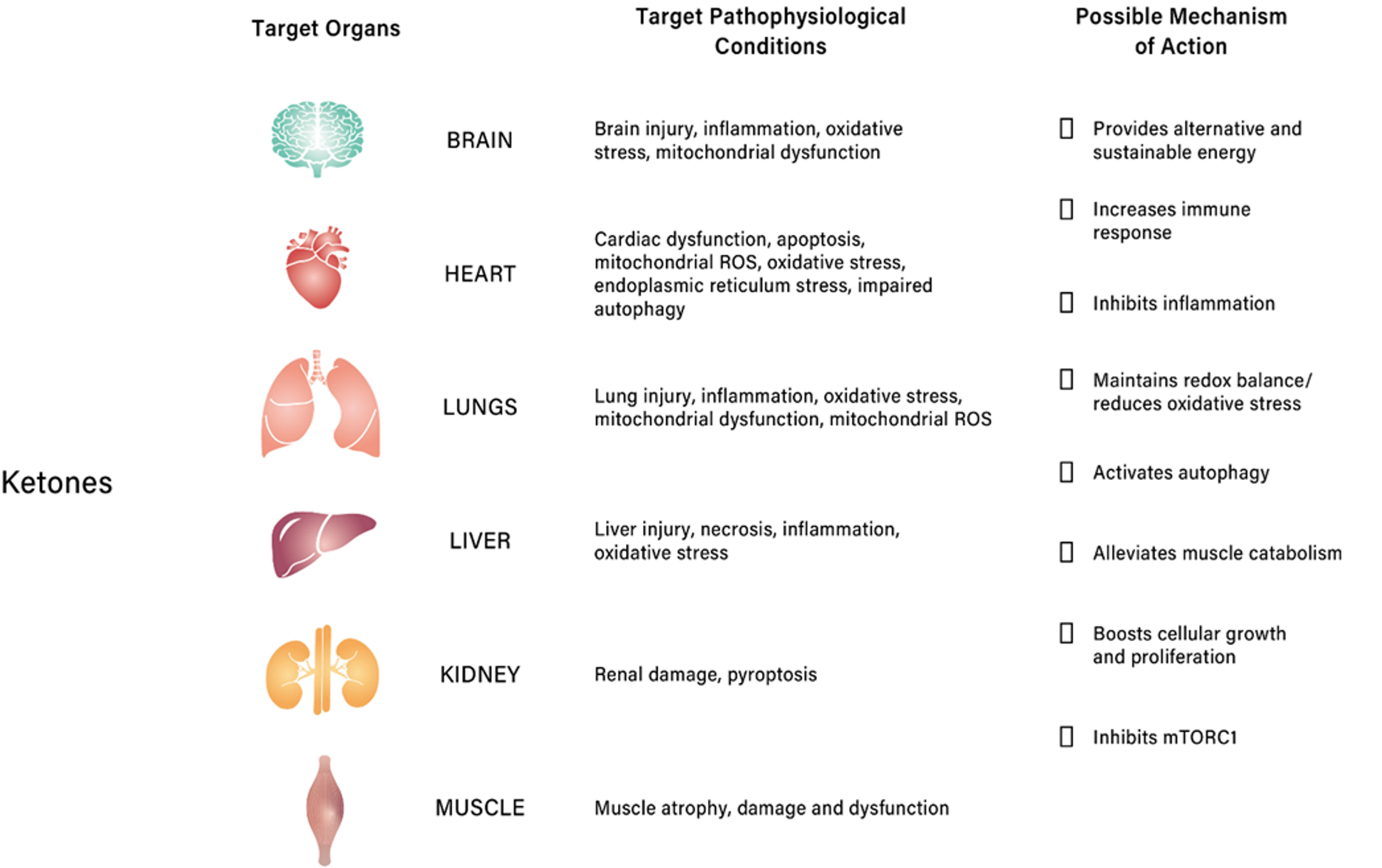

Supplement: Supplementary file 1 [file nutrients-14-03613-s001.zip › nutrients-1880428-supplementary.tif]
